# Can ivermectin mass drug administrations to control scabies also reduce skin and soft tissue infections? Hospitalizations and primary care presentations lower after a large-scale trial in Fiji

**DOI:** 10.1016/j.lanwpc.2022.100454

**Published:** 2022-04-11

**Authors:** Jo Middleton

**Affiliations:** a NIHR Global Health Research Unit on Neglected Tropical Diseases and Department of Primary Care and Public Health, Brighton and Sussex Medical School, Falmer, United Kingdom; b Evolution, Behavior and Environment, School of Life Sciences, University of Sussex, Falmer, East Sussex, United Kingdom

Evaluations of Mass Drug Administrations (MDAs) targeting scabies have generally not assessed their secondary impacts on skin and soft tissue infections. These are major health burdens in the global south, with sequelae including glomerulonephritis, rheumatic fever and chronic kidney disease.^1^, ^2^, ^3^ In a major step forward Li Jun Thean and colleagues^4^ report in *The Lancet Regional Health Western Pacific* on the impact on skin infections of an oral ivermectin MDA targeting scabies in Fiji. Importantly, they show in the largest before-after trial of its kind that not only did scabies and impetigo prevalence reduce, but so too did hospitalisations with severe skin and soft tissue infections and attendance at primary care with scabies and skin infections.

Scabies is a highly irritant skin infestation with the mite *Sarcoptes scabiei* and is primarily transmitted by touch. It is classed by WHO as a Neglected Tropical Disease (NTD) in recognition of its high burden and the health risks associated with secondary infections of impetigo (i.e., cellulitis, necrotising fascittis, skin abscesses, rheumatic fever, chronic kidney disease).^1^, ^2^, ^3^ Evidence indicates prevalence is not influenced by personal washing.^5^ Neglected tropical skin diseases are significant health problems across Oceania.^6^^,^^7^ Some of the world's highest prevalence rates of scabies have been measured in the region (e.g., Papua New Guinea, 71%; Fiji, 32%).^2^ The situation is similar for impetigo, particularly in children (e.g., Samoa, 57%; Solomon Islands, 52%; Fiji, 36%; Vanuatu, 16%).^2^^,^^8^ The first trial in Oceania of an ivermectin MDA for scabies (outside of institutional outbreaks) was carried out in a Papua New Guinea village in 1996,^9^ followed on a greater scale in the Solomon Islands 1997–2000,^10^ Fiji in 2004,^11^ and in aboriginal communities in Australia.^12^ A 15-year follow-up of the first Solomon Islands MDA found very low scabies prevalence, in fact just one case. Marks et al.^10^ attributed this to MDA and an associated period of active case finding. Subsequently, in the Solomon Islands^13^ and Fiji^14^ MDAs targeting scabies have been associated with decreased impetigo prevalence (respectively: from 24.7% to 9.6%; 24.6% to 8.0%). Influenced by these developments in Oceania, similar interventions are increasingly being conducted elsewhere.^1^ The largest adoption of this approach has been in Ethiopia where a 2018 scabies MDA involved over nine million people,^15^ and the secondary impacts on scabies of an ivermectin MDA targeting onchocerciasis is also being assessed.^16^

Against this regional and global background Thean and colleagues^4^ report a 2018–2020 before-after MDA trial covering Fiji's Northern Division: first dose, 135,744 people (97% coverage); second dose, 121,760 people (87%). The prior MDA in Fiji had 716 participants enrolled in the ivermectin arm,^14^ in the Solomon Islands 26,188.^13^ This then is by far the largest study to date to report the impact of a scabies MDA on impetigo. Scabies prevalence decreased from 14.2% to 7.7%, and impetigo from 15.3% to 6.1%. Whilst this valuably supports the conclusions of the earlier smaller trials, what is most important in this study is the observed reductions in primary and secondary care burdens. Incidence of childhood invasive infections and post-streptococcal sequelae were unchanged. However, hospitalisations with severe skin and soft tissue infections reduced 17%, attendance at primary care with scabies and skin infections 21%. Thean and colleagues note the reduction in scabies prevalence was lower than expected compared to other in-region studies. They are likely correct in linking this in part to the intervention not being distributed and overseen directly by the study team, as many smaller scabies MDA trials have been, but instead by the national health system. A similar reduction in expected effectiveness has been observed in a non-researcher run MDA in Ethiopia.^16^ Rather than a weakness of the study, this underlines its value as the important effects shown may more realistically indicate benefits to be expected when operationalising MDAs at large-scales beyond smaller researcher-run trials. These results strongly support (1) expansion of ivermectin MDAs targeting scabies and impetigo, (2) collection of similar hospitalisation and primary care data as MDAs expand, and (3) development of further research and interventions that capitalise on how NTD control can secondarily reduce wider burdens to health systems. Given resource scarcity such synergistic integrations are important clinically not only in Oceania^6^, but worldwide.^1^

## Declaration of interests

The author declares that they have no competing interests.
